# Comorbidity in borderline personality disorder and adult attention deficit hyperactivity disorder in the context of impulsivity and emotional dysregulation

**DOI:** 10.1192/j.eurpsy.2022.1167

**Published:** 2022-09-01

**Authors:** R. Tóth, E. Kenézlői, B. Bajzát, L. Balogh, S. Somogyi, Z. Unoka, J. Réthelyi

**Affiliations:** 1 Hungarian Association for Behavioural, Cognitive and Schema Therapies, Psychology, Budapest, Hungary; 2 Semmelweis University, Department Of Psychiatry And Psychotherapy, Budapest, Hungary

**Keywords:** comorbidity, ADULT ADHD, Impulsivity, BPD

## Abstract

**Introduction:**

In a significant proportion of people diagnosed with Borderline Personality Disorder (BPD) and adult Attention Deficit Hyperactivity Disorder (aADHD) comorbid mental disorders, such as mood, anxiety, personality and substance use disorders can be detected. BPD and aADHD present with a partial overlap in the clinical symptoms, including increased impulsivity levels and difficulty in emotional regulation. Higher impulsivity and emotional dysregulation (ED) can result in impaired global functioning or damaged social relationships.

**Objectives:**

The aim of this study was to assess the comorbid psychiatric diagnoses of the two patient groups, and explore the possible role of ED and impulsivity in the background of the different comorbid disorders.

**Methods:**

Data about BPD (N=49) and aADHD (N=60) patients were analyzed based on the M.I.N.I. Plus 5.0 and SCID-5-PD structured clinical interviews. Participants were further investigated with online questionnaires: e.g. Barratt Impulsiveness Scale (BIS-11) Difficulties in Emotion Regulation Scale (DERS). For measuring the influence of impulsivity and ED in the development of comorbid disorders binary logistic regression was used.

**Results:**

Our results showed comorbidity rates similar to previous findings in BPD patients, but lower rates were observed in aADHD. Elevated levels of ED increases the risk of suicidal ideation, mood, anxiety and eating disorders. Based on our data increased impulsivity can reduce the chance of comorbid anxiety disorders.

**Conclusions:**

The results provide insight into the pattern of comorbid disorders, role of ED and impulsivity in people diagnosed with aADHD and BPD in Hungary. Understanding their underlying mechanisms helps to establish an accurate diagnosis, which affects treatment effectiveness.

**Disclosure:**

No significant relationships.

